# An Evaluation of the Relationship between Body Mass Index (BMI), Dietary Habits, and the Prevalence of Dental Caries in Children Aged 4 to 12

**DOI:** 10.3290/j.ohpd.c_1896

**Published:** 2025-03-06

**Authors:** Mihriban Gökcek Taraç, Taibe Tokgöz Kaplan

**Affiliations:** a Mihriban Gökcek Taraç Assistant Professor, Department of Pediatric Dentistry, Karabük University School of Dentistry, Karabük, Turkey. Concept, design, supervision, resources, data collection, analysis and interpretation, writing manuscript, critical review, approval.; b Taibe Tokgöz Kaplan Assistant Professor, Department of Pediatric Dentistry, Karabük University School of Dentistry, Karabük, Turkey. Concept, design, resources, materials, data collection, literature search, writing manuscript, critical review, approval.

**Keywords:** body mass index, dental caries, obesity

## Abstract

**Aim:**

To evaluate the relationship between body mass index (BMI) and dental caries in children aged 4–12 years.

**Materials and Methods:**

367 children referred to the pedodontics clinic were included in our study. In this two-stage study, firstly the decayed, missing, and filled teeth index (DMFT) or dental caries index (dft) scores of the children were recorded by oral examination, and their weight and height measured. Secondly, the children’s parents or legal representatives were asked to complete a questionnaire assessing sociodemographic data and their children’s nutritional habits. The data obtained were analysed statistically. In multiple comparisons of variables showing continuous variation with normal distribution, ANO-VA post-hoc analysis and Tukey’s tests were used. For variables not showing normal distribution, Kruskal–Wallis post-hoc analysis and Mann–Whitney U tests were used.

**Results:**

Considering their BMI, 34.1% children were underweight, 30.8% were of normal weight, 14.4% were overweight, and 20.7% were obese. A significant relationship was found between the children’s BMI and age groups (P = 0.000) and BMI increased as age decreased. Both BMI and dental caries incidence increased as the consumption of carbohydrates and sugar products increased. The mean DMFT score was higher for children with overweight BMI. The mean dft score was higher among children in the obese BMI category.

**Conclusion:**

High BMI and dental caries are multifactorial disorders with similar risk factors, and the relationship between both is still not fully clear in the literature. Although cross-sectional studies provide the infrastructure for future studies by revealing the prevalence of the disorder, risk factors, and possible consequences, they are inadequate to examine the cause–effect relationship. More detailed and longer-term studies are needed to establish the causal relationship between BMI and dental caries.

Dental caries are one of the most prevalent chronic diseases in children.^7,40^ Although it is claimed that the incidence of dental caries might decrease in high-income countries with the widespread use of preventive dental practices and easier access to dental services, rapid urbanisation, industrialisation, economic development, and lifestyle and nutritional habit changes make it difficult to control dental caries.^32,33^ The relationship between refined carbohydrate consumption and dental caries also extends to obesity.^19^ Obesity is a health problem affecting 650 million people worldwide^38^ and affects both the general health and cognitive development of children.^35^ Various predisposing factors, such as lifestyle, biological, genetic, cultural, socioeconomic, environmental, and dietary influences are common in the aetiology of both obesity and dental caries.^6,30^ Eating habits, spending too much time watching television or consuming social media, along with a decrease in physical activity and unhealthy snack intake during this time contribute to the development of obesity and dental caries.^8,18^

Various studies have been conducted examining the relationship between dental caries and body mass index (BMI), but no definitive consensus has been reached from these studies.^12^ While some researchers state that the incidence of tooth decay is high in individuals with a high BMI,^14,21^ others have found no significant correlation.^3,9^ However, Mishu et al have reported that weight loss might be normal in children with severe dental caries and that low BMI could be associated with a high prevalence of dental caries.^22^ In addition to being positively associated with tooth decay, obesity is a serious condition that can lead to deterioration of general health in children, including high blood pressure, diabetes, and cardiovascular problems if not controlled.^31^ Despite various efforts of the World Health Organization against obesity, the increase in unhealthy dietary habits increases both dental caries and obesity in children.^2^ In addition, not fully understanding the relationship between obesity and tooth decay prevents effective treatment of these diseases. Therefore, this study aimed to evaluate the relationship between BMI and dental caries in children aged 4–12 years.

## MATERIALS AND METHODS

### Study Design

This study was a descriptive, cross-sectional study that included 367 children who were referred to the Karabük Oral and Dental Health Training and Research Hospital in Turkey. Ethical approval was obtained from Karabük University (protocol number: 2020/326). Data were collected from participants who were seeking treatment the pedodontics clinic between July 2022 and September 2023. Parents or legal caregivers were informed of the study and consent forms were obtained.

### Hypothesis

Higher BMI and poor nutritional habits are positively correlated with an increased prevalence of dental caries in children aged 4–12 years.

### Sample Size

The sample size was calculated based on a previous study assessing the relationship between dental caries and BMI.^24^ Considering a 0.05 alpha value and 95% power, the minimum sample size was calculated as 346 children. The number of samples was determined as 400 in case there were incompletely filled out surveys. When the incompletely filled out survey forms were removed, a total of 367 people remained for the study.

### Inclusion and Exclusion Criteria

The inclusion criteria were that the children were aged 4–12 years, allowed intraoral examination, and that the families were willing to complete the survey. The exclusion criteria were if children were uncooperative and did not allow intraoral examination; they had chronic diseases, physical deformities, or mental illness; were undergoing orthodontic treatment; or if children and their parents refused to participate in the study.

### Data Collection

Our study consisted of two stages. In the first stage, parents were asked to complete a questionnaire. In the second stage, the children’s oral examination was performed, and their body weight and height were measured. The first part of the questionnaire contains sociodemographic information about the children, while the second part contains information about their nutritional habits. The third part of the questionnaire contains questions about the parents’ socioeconomic status and oral hygiene habits. The first three parts of the questionnaire were filled out by parents. In the fourth section, there is a table where decayed, missing, and filled teeth in primary and permanent teeth were marked. The fifth section includes spaces where body weight and height are noted. These sections were filled in by the researchers. The intraoral examination was performed by two trained and calibrated dentists. Before the examination, the children were asked to remove food debris by rinsing their mouths. The examinations were performed identically for each child, starting from the right upper quadrant and proceeding clockwise. Tooth surfaces were evaluated under visible light using a mirror and a round-tipped probe. Each tooth was scored according to whether they were decayed, missing, or filled. DMFT indexes were calculated for permanent teeth and dft indexes were calculated for primary teeth according to World Health Organization methods. The sum of the obtained values indicates the history of dental caries. The bodyweights of the participants were measured without shoes and height was measured using a stadiometer while standing upright. BMI was evaluated by dividing the weight (kg) by the square of the height (m²). All children were categorised based on the BMI score as follows: underweight ( <18.50 kg/m²), normal weight (18.50–24.9 kg/m²), overweight (25.00–30.99 kg/m²), and obese (> 30.00 kg/m²).^37^

### Statistical Analysis

Statistical analyses of our study were performed using the MiniTab 17 Statistics Program (Statistical Software Release, Version 17.3.1, Minitab, USA). For continuous variables, the mean and standard deviation were calculated, while for categorical variables, frequency and percentage values were reported. For multiple comparisons of variables showing continuous variation with a normal distribution, ANOVA post-hoc analysis and Tukey’s tests were used. For variables not showing a normal distribution, Kruskal–Wallis post-hoc analysis and Mann–Whitney U tests were applied. A statistical significance level of P < 0.05 was used for all tests.

## RESULTS

### Study Group Profile

Of the 367 children included in our study, 51.2% (n = 188) were girls and 48.8% (n = 179) boys and their mean age was 7.9 ± 2.14 years. Based on their BMI, 34.1% were classed as underweight, 30.8% had a normal weight, 14.4% were overweight, and 20.7% were obese. General information about the participants and their families are provided in Table 1.

**Table 1:** Characteristics of participants

Variables	N	%
Age
4–6 years	115	31.4
7–9 years	159	43.3
10–12 years	93	25.3
Brushing frequency
Sometimes	115	31.3
Once in a day	167	45.5
Twice or more in a day	85	23.2
BMI
Underweight	125	34.1
Normal	113	30.8
Overweight	53	14.4
Obese	76	20.7
Number of children
1	47	12.8
2	162	44.1
>2	158	43.1
Monthly income
≤ Minimum wage	144	39.2
Minimum wage – 30.000 TL	127	34.6
>30,000 TL	96	26.2
Education level of parents
Primary school	123	33.5
High school	134	36.5
University and above	110	30
Brushing frequency of parents
Sometimes	65	17.7
Once in a day	192	52.3
Twice or more in a day	110	30.0


A statistically significant relationship was found between the children’s BMI and age groups (P = 0.04). Specifically, obesity was more common in younger age groups and children tended to have a normal BMI as they got older. Additionally, a statistically significant relationship was found between BMI and the educational status of the families, with obesity rates increasing with higher education level of their families. However, there was no statistically significantt relationship between BMI and sex, the number of children in the family, or the monthly family income (Table 2).

**Table 2:** BMI and demographic characteristics

	N	BMI	P value
Underweight	Normal weight	Overweight	Obese	Total
N	%	N	%	N	%	N	%	N	%
**Age**	4–6 years	47	40.9	21	18.3	12	10.4	35	30.4	115	100	**0.04**
7–9 years	56	35.2	55	34.6	22	13.8	26	16.4	159	100
10–12 years	22	23.7	37	39.8	19	20.4	15	16.1	93	100
**Sex**	Female	72	38.3	57	30.3	23	12.2	36	19.2	188	100	**0.612**
Male	53	29.6	56	31.3	30	16.8	40	22.3	179	100
**Brushing frequency**	Sometimes	35	30.4	34	29.6	20	17.4	26	22.6	115	100	**0.469**
Once in a day	62	37.1	45	26.9	22	13.2	38	22.8	167	100
Twice or more in a day	28	32.9	34	40	11	12.9	12	14.2	85	100
**Monthly income**	≤minimum wage	45	31.3	48	33.3	16	11.1	35	24.3	144	100	**0.343**
Minimum wage–30.000 TL	47	37	37	29.1	16	12.6	27	21.3	127	100
>30.000	33	34.4	28	29.1	21	21.9	14	14.6	96	100
**Education level of parents**	Primary school	41	33.3	56	45.5	18	14.7	8	6.5	123	100	**0.02**
High school	60	44.8	21	15.7	27	20.1	26	19.4	134	100
University and above	24	21.8	36	32.7	8	7.3	42	38.2	110	100


### Dietary and Lifestyle Habits

The frequency of participants’ consumption of carbohydrates, acidic foods, sugary foods, and dairy products was evaluated. No statistically significant relationship was found between bottle use (P = 0.670), duration of bottle use (P = 0.831), frequency of consumption of acidic foods (P = 0.723), and children’s sports habits (P = 0.459) and BMI. As carbohydrate intake decreased, the rate of children with a normal BMI increased, whereas an increase in carbohydrate intake generally corresponds to a higher BMI. Additionally, a statistically significant relationship was found between sugar and dairy product consumption and BMI (Table 3).

**Table 3:** Dietary habits and BMI

		BMI	P value
Underweight	Normal weight	Overweight	Obese	Total
N	%	N	%	N	%	N	%	N	%
**Breastfeeding**	0–6 months	31	34.8	14	15.7	16	18.0	28	31.5	89	100	**0.01**
7–12 months	26	54.2	4	8.3	13	27.1	5	10.4	48	100
More than 1 year	68	29.6	95	41.3	24	10.4	43	18.7	230	100
**Carbohydrate intake**	Twice or less in a week	28	40.6	31	44.9	6	8.7	4	5.8	69	100	**0.001**
3 or more in a week	72	32.3	75	33.6	39	17.5	37	16.6	223	100
Everyday	25	33.3	7	9.3	8	10.7	35	46.7	75	100
**Sugar intake**	Twice or less in a week	64	51.6	38	30.7	9	7.2	13	10.5	124	100	**0.03**
3 or more in a week	53	29.6	54	30.2	30	16.8	42	23.4	179	100
Everyday	8	12.5	21	32.8	14	21.9	21	32.8	64	100
**Dairy products intake**	Twice or less in a week	21	58.3	2	5.6	2	5.6	11	30.5	36	100	**0.01**
3 or more in a week	16	11.3	46	32.6	38	27.0	41	29.1	141	100
Everyday	88	46.3	65	34.2	13	6.9	24	12.6	190	100


### DMFT/dft Index and BMI

The mean DMFT value of the children was 1.2 (±1.93) and mean dft value was 6.1 (±4.09). The highest dft value was observed in the age 4–6 year group, while the highest DMFT value was seen in the age 10–12 year group. The relationship between DMFT and dft scores with BMI is given in Table 4. The mean DMFT score was higher among the overweight BMI category, while the mean dft score was higher among the obese BMI category. These differences were found to be statistically significant.

**Table 4:** DMFT/dtf scores and BMI

Variables	BMI	Mean	Standard deviation	P value
**dft**	Underweight	6.43	3.96	**0.001**
Normal weight	5.42	4.21
Overweight	5.11	4.08
Obese	7.32	3.79
**DMFT**	Underweight	1.09	2.06	**0.0008**
Normal weight	1.43	1.99
Overweight	1.51	2.18
Obese	0.85	1.29


There was no significant relationship between breastfeeding duration, nursing bottle use, intake of acidic foods, and dairy products with dft/DMFT values. However, a statistically significant relationship was found between the frequency of carbohydrate intake and sugar consumption with dental caries (Table 5).

**Table 5:** Dietary habits and dental caries

Variables	N	%	dft	DMFT
**Breastfeeding**
0–6 month	89	24.2	6.1	1.3
7–12 month	48	13.1	5.3	1
More than 1 year	230	62.7	6.3	1.2
**P value**	**0.17**	**0.51**
**Nursing bottle use**
Yes	157	42.8	5.9	1.3
No	210	57.2	6.3	1.2
**P value**	**0.31**	**0.46**
**Carbohydrate intake**
Twice or less in a week	69	18.8	5	2.1
3 or more in a week	223	60.8	6.7	0.1
Everyday	75	20.4	5.4	1.1
P value	0.02	0.01
**Acidic food intake**
Twice or less in a week	291	79.3	6.1	1.1
3 or more in a week	68	18.5	6.2	1.4
Everyday	8	2.2	6.3	1.6
**P value**	**0.17**	**0.43**
**Sugar intake**
Twice or less in a week	124	33.8	5.4	1.7
3 or more in a week	179	48.8	6.3	1.0
Everyday	64	17.4	7.1	0.7
**P value**	**0.002**	**0.04**
**Dairy products**
Twice or less in a week	36	9.8	6.4	1.5
3 or more in a week	141	38.4	6.2	1.4
Everyday	190	51.8	5.9	1.1
**P value**	**0.07**	**0.42**


## DISCUSSION

In our study, a statistically significant relationship was observed between the consumption of sugary foods and carbohydrates and BMI. Frequent consumption of these foods, which also play a role in the aetiology of dental caries, increased the incidence of dental caries. From this perspective, the aetiology of obesity and overweight was similar to the aetiology of dental caries and there was a positive correlation between BMI and the incidence of dental caries.

The aetiology of both obesity and dental caries appear to be influenced by multiple factors, such as socioeconomic status, lifestyle, education level, dietary habits, and psychosocial behaviours. Poor dietary habits, particularly an increased intake of sugar, are significant risk factors for both obesity and dental caries. When reviewing the literature, although some studies detect a strong positive relationship between obesity and dental caries,^15,28^ there are also studies indicating a negative correlation between increasing BMI and the incidence of dental caries.^12,13^ Therefore, new studies in different regions are needed to evaluate the relationship between obesity and dental caries. For this purpose, our study assessed the relationship between BMI and dental caries in children aged 4–12 years.

In 2012, the Organization for Economic Cooperation and Development reported the frequency of overweight (including obesity) among children aged 5–17 years in Turkey as 11.3% in boys and 10.3% in girls.^36^ According to the updated data from the Turkish Statistical Institute in 2022, 23.6% of women are classified as obese and 30.9% as pre-obese, while 16.8% of men are obese and 40.4% are pre-obese in individuals aged 15 years and older.^34^ In our study, 34.1% of participants were underweight, 30.8% had a normal weight, 14.4% were overweight, and 20.7% were obese. Similar to our study, a study in Riyadh, Saudi Arabia reported the incidence of children with obesity as 19.0%.^16^ It is thought that the global pandemic has contributed to the increasing incidence of obesity by causing a significant shift in individuals’ active lifestyles, leading to a more sedentary and unhealthy way of living.^1^

In our study, the prevalence of obesity was higher in children in the younger age group. In a study conducted by Mahmoud et al, on patients aged 5–9 years, the obesity rate was 66.9%.^20^ It is thought that the higher obesity rate in the younger age group, which tends to normalise as they grow older, is related to the changing eating habits of children.

Although it is known that increased cortisol levels, stress, and anxiety in women during puberty predispose them to weight gain,^4^ our study found that high BMI and obesity were more common in men than in women. However, the relationship between sex and BMI was not statistically significant. Similarly, Mahmoud et al reported a higher obesity rate in men.^20^

The increase in childhood obesity can be attributed to sedentary lifestyles, unhealthy diets, and decreased physical activity among children in high-income families from economically well-off countries.^25^ The relationship between socioeconomic status and obesity is generally complex. There are studies indicating that the incidence of malnutrition and therefore low BMI is higher in underdeveloped countries.^1^ It has also been observed that in underdeveloped countries, the frequency of obesity increases due to high-calorie diets and low physical activity among children from high-income families. Conversely, in developed countries, the incidence of obesity tends to be lower because of high public awareness regarding nutritional habits among families with high incomes.^17^ In our study, obesity decreased in individuals with high monthly income, but this finding was not statistically significant. Additionally, a statistically significant relationship was found between the education level of the families and BMI, with a higher incidence of obesity noted among children of parents who were university graduates.

In our study, similar to the findings of Alghamdi et al, it was observed that the frequency of caries in primary dentition was higher in children who were obese, while the frequency of caries in permanent dentition was higher in those who were of normal weight or overweight.^1^ The prevalence of obesity was higher in the age 4–6 year group, where primary dentition is prevalent. This high number of obese children in the age 4–6 year group explains the increased dft scores in children with obesity.

Previous studies have shown that increased BMI is generally associated with a high frequency of carbohydrate intake, which in turn contributes to dental caries.^14^ Similarly, in our study, a statistically significant relationship was observed between carbohydrate intake, BMI, and DMFT and dft values. Ruottinen et al reported that high amounts of sugar intake in children from infancy to age 10 years led to increased BMI and tooth decay.^27^ In our study, a statistically significant relationship was found between sugar consumption, BMI, and dental caries.

Low BMI can be associated with malnutrition, which might lead to hypoplasia of teeth, hypofunction of salivary glands, and changes in the composition of saliva, all of which can contribute to dental caries. Conversely, severe dental caries and cavitations might hinder children eating properly, potentially resulting in low body weight.^29^ It is possible that sleep disturbances caused by toothaches could also contribute to weight loss.^23^

Low socioeconomic status and lower education levels of families can reduce access to healthy nutrition, leading to deviations from normal BMI and increased incidence of tooth decay.^21^ Poor oral hygiene can prevent children from obtaining adequate nutrition, leading to a decrease in BMI and negatively affecting the child’s growth and cognitive development.^5^

In studies that claim there is no relationship between obesity and dental caries, it has been stated that the frequency of sugar intake is more important than the total amount of sugar consumed. Even small amounts of sugar consumed frequently can lead to dental caries without necessarily causing a change in BMI.^10^

Studies evaluating the relationship between BMI and dental caries give contradictory results. The main reason for these contradictory results is thought to be the non-linear relationship between dental caries and BMI. Dental caries are more common in individuals with both high and low BMI.^39^ Similarly, in our study, dft values were highest in both the underweight and obese groups. Ravelomantsoa et al., also reported a relationship between dental caries and both high and low BMI.^26^

## CONCLUSION

Although no definitive conclusion can be reached from the studies conducted, understanding the factors affecting BMI and evaluating the relationship between increasing BMI and the incidence of dental caries are important for informing the public and developing preventive programmes. Consuming high amounts of sugar is a modifiable behaviour that affects both BMI and dental caries. Therefore, studies on obesity and dental caries should clearly identify risk factors and investigate ways to prevent them.

While cross-sectional studies, such as this, provide the foundation for future research by revealing the prevalence of the disorder, risk factors, and possible consequences, they are inadequate for examining the situation in a cause–effect relationship. More detailed and longer-term studies are needed to establish the causal relationship between BMI and dental caries, and to provide effective interventions for these variables. Additionally, our study can be considered a pilot study due to the limited number of participants. A more comprehensive evaluation of the relationship between BMI and dental caries could be achieved with larger-scale studies.

### Acknowledgement

We extend our heartfelt gratitude to all the patients and their parents who graciously participated in this survey. Their collective efforts and support were invaluable in making this study possible.

### Ethics Committee Approval

Karabük University Ethical Committee/Protocol No: 2020/326.

### Informed Consent

Verbal consent for intraoral examination was obtained from the children participating in the study, and written consent was obtained from their legal guardians on behalf of both themselves and their children.
